# Accuracy of residential geocoding in the Agricultural Health Study

**DOI:** 10.1186/1476-072X-13-37

**Published:** 2014-10-07

**Authors:** Rena R Jones, Curt T DellaValle, Abigail R Flory, Alex Nordan, Jane A Hoppin, Jonathan N Hofmann, Honglei Chen, James Giglierano, Charles F Lynch, Laura E Beane Freeman, Gerard Rushton, Mary H Ward

**Affiliations:** Occupational and Environmental Epidemiology Branch, Division of Cancer Epidemiology and Genetics, National Cancer Institute, National Institutes of Health, Department of Health and Human Services, 9609 Medical Center Drive, Rockville, MD USA; Westat Inc., Rockville, MD USA; Department of Geographical and Sustainability Sciences, University of Iowa, Iowa City, IA USA; Department of Biological Sciences, North Carolina State University, Raleigh, NC USA; Epidemiology Branch, National Institute of Environmental Health Sciences, National Institutes of Health, Department of Health and Human Services, Research Triangle Park, Durham, NC USA; Iowa Department of Natural Resources, Iowa Geological and Water Survey, Iowa City, IA USA; Department of Epidemiology, College of Public Health, University of Iowa, Iowa City, IA USA

**Keywords:** Geocoding, Positional error, Rural location, Environmental exposure assessment, Accuracy

## Abstract

**Background:**

Environmental exposure assessments often require a study participant’s residential location, but the positional accuracy of geocoding varies by method and the rural status of an address. We evaluated geocoding error in the Agricultural Health Study (AHS), a cohort of pesticide applicators and their spouses in Iowa and North Carolina, U.S.A.

**Methods:**

For 5,064 AHS addresses in Iowa, we compared rooftop coordinates as a gold standard to two alternate locations: 1) E911 locations (intersection of the private and public road), and 2) geocodes generated by matching addresses to a commercial street database (NAVTEQ) or placed manually. Positional error (distance in meters (m) from the rooftop) was assessed overall and separately for addresses inside (non-rural) or outside town boundaries (rural). We estimated the sensitivity and specificity of proximity-based exposures (crops, animal feeding operations (AFOs)) and the attenuation in odds ratios (ORs) for a hypothetical nested case–control study. We also evaluated geocoding errors within two AHS subcohorts in Iowa and North Carolina by comparing them to GPS points taken at residences.

**Results:**

Nearly two-thirds of the addresses represented rural locations. Compared to the rooftop gold standard, E911 locations were more accurate overall than address-matched geocodes (median error 39 and 90 m, respectively). Rural addresses generally had greater error than non-rural addresses, although errors were smaller for E911 locations. For highly prevalent crops within 500 m (>97% of homes), sensitivity was >95% using both data sources; however, lower specificities with address-matched geocodes (more common for rural addresses) led to substantial attenuation of ORs (e.g., corn <500 m OR_obs_ = 1.47 vs. OR_true_ = 2.0). Error in the address-matched geocodes resulted in even greater OR_obs_ attenuation for AFO exposures. Errors for North Carolina addresses were generally smaller than those in Iowa.

**Conclusions:**

Geocoding error can be minimized when known coordinates are available to test alternative data and methods. Our assessment suggests that where E911 locations are available, they offer an improvement upon address-matched geocodes for rural addresses. Exposure misclassification resulting from positional error is dependent on the geographic database, geocoding method, and the prevalence of exposure.

**Electronic supplementary material:**

The online version of this article (doi:10.1186/1476-072X-13-37) contains supplementary material, which is available to authorized users.

## Background

Identifying the geographic location of individuals over time and space is a critical step in the analysis of spatial clustering of disease risk and in linking environmental contaminants with human health risks. This location must be highly accurate in order to conduct exposure assessments that correctly represent exposure to individuals, including proximity to pollution sources.

Though resource-intensive, two highly accurate methods are often available to identify a location. The first is to use a global positioning system (GPS) to obtain geographic coordinates for a study participant’s residence; the second is to locate the residence on a registered orthophoto image upon which individual residential addresses have been identified. However, this latter method is impractical if the interview is not conducted at the home of interest, which is the case for historical residences or if in-person interviews are not conducted. In these instances, residential addresses are usually geocoded by matching addresses to a geo-referenced street file in a Geographic Information System (GIS). These reference databases include the spatial location of streets and intersections, often developed or enhanced by city and county governments using local geographic and administrative data. The basic geocoding operation is to interpolate an address location along a street segment (i.e., the section of a street between intersections with known coordinates). The address is located proportional to its street number within the range of numbers for the street segment [1]. Typically, coordinates are assigned to the address after applying a fixed distance offset from the street centerline. Geocoded locations are then used to estimate residential proximity to a pollution source or some other spatially-derived metric of exposure, such as estimates of air quality.

A limitation of this approach is the known heterogeneity in the positional accuracy of geocoded addresses, with greater errors consistently observed in rural compared to non-rural areas [2–4]. These larger errors may be the consequence of unique features of rural addresses, such as long driveways that result in significant differences between the true residential location and the street-based geocode, or rural route addresses that do not exist in commercial street databases used for geocoding. Without high quality location information, geocodes are sometimes limited to the zip code centroid or some other coarse resolution surrogate for the actual spatial position. This positional error may result in exposure misclassification and potentially biased risk estimates in an epidemiologic analysis, with consequences that may vary by the magnitude of error and spatial scale of the exposure data [5–7].

The agricultural setting includes numerous environmental exposures which may be assessed using GIS methods. For example, farmers and their families may be exposed to pesticides, fertilizers, and animals both occupationally and as a consequence of their workplace also being their residential environment. Other rural residents may experience farming-related exposures through their close proximity. The Agricultural Health Study (AHS), a large prospective cohort of private pesticide applicators in Iowa and North Carolina, U.S.A., their spouses, and commercial pesticide applicators in Iowa [8], was designed to examine such exposures to farmers and to their families. Residential addresses reported at enrollment and two follow-up interviews have been geocoded as the first step in assessing indirect (bystander) exposure to agricultural pesticides and to estimate contaminants in their private wells. Because locational errors have implications for exposure assessment, and most AHS participants (73%) resided in rural areas at enrollment, it is important to characterize this error and the potential for exposure misclassification in epidemiologic analyses of AHS data. Moreover, such an assessment may also be applicable to epidemiologic studies of rural populations worldwide, although the sources and extent of error in geocoding may vary by geographic region or country. The objective of this study was to determine the positional error of a sample of AHS participant addresses located using two data sources (address-matched geocodes and E911 locations) and to evaluate the effect of the location errors on the validity of estimates of agricultural exposures.

## Methods

### Study population

Details of the AHS, including the study design and a description of the cohort, have been previously published [8]. Initiated in 1993, the AHS is an ongoing prospective cohort of 52,394 licensed private pesticide applicators (primarily farmers) and 32,346 of their spouses residing in two U.S. states, Iowa and North Carolina, and 4,916 licensed commercial pesticide applicators in Iowa. Pesticide applicators were identified while applying or training for restricted-use pesticide licenses, at which time they were asked to complete an enrollment questionnaire. Private applicators’ spouses were enrolled by completing a take-home questionnaire given to the applicator and returned by mail. Two follow-up surveys of private applicators and their spouses have been conducted via computer-assisted telephone interviews. The address prior to the enrollment address was obtained from spouses in the initial questionnaire. The present assessment focuses on all addresses for private applicators and spouses in 14 Iowa counties. These counties were selected based on the availability of county-wide reference geo-locations (rooftop coordinates and E911 locations, described in detail below) and tended to be in the North-central area of Iowa (Additional file 1: Figure S1).

We also included address data from two AHS ancillary studies for which GPS readings were taken as part of an in-home interview: the Biomarkers of Exposures and Effects in Agriculture (BEEA) study and the Lung Health (LH) study. Addresses for both studies were located throughout Iowa and North Carolina, allowing for within-state and between-state comparisons.

### Reference geo-locations

Rooftop coordinates for all addresses in 14 counties in Iowa were supplied by the Iowa Geological and Water Survey (IGWS) from their statewide geocoding project. Rooftop coordinates were placed in the center of all identified structures using 2010 National Agriculture Imagery Program (NAIP) orthophotos (aerial images with 1-meter pixel resolution, registered to Universal Transverse Mercator coordinates) as a reference base map. This was accomplished by making a visual interpretation of each structure using the 2010 NAIP orthophoto and checking with county assessor tax assessment web sites for land use categories, floor plans, and other real estate information about the property. County GIS parcel data were then used to assign address attributes (house number, street, direction, city) to the structure points falling within each property boundary. Address attributes were then standardized using commercial, U.S. Postal Service ZIP + 4® addressing software. U.S. Census 2000 Incorporated Places boundary data were used to classify residences as being located as within (non-rural) or outside an incorporated place (rural), determined by the rooftop coordinates.

GPS data for the two AHS subcohorts were collected as part of their individual study protocols. Lung Health GPS data were collected between February 2009 and May 2012 near the main entrance of participant residences using a Garmin® GPSMAP 60Cx Navigator. GPS data were reviewed when the GPS location was more than 1000 m from the geocoded street address. Incorrect GPS readings due to equipment and/or operator error (5.5%), visits entirely or partially at non-residence locations (0.8%), and participants with P.O. Box addresses (0.3%) were excluded from our analyses. GPS data for the BEEA study were collected between June 2010 and April 2013 near the main entrance of participant residences using a Garmin® 76CSX handheld GPS device. A similar quality assessment led to exclusions from the analysis for incorrect GPS readings due to equipment and/or operator error (<1%) or if the GPS reading was not located in the correct city (<1%).

### E911 locations

As part of a U.S. national effort to improve emergency response, most rural route addresses have been converted to street addresses. For many counties, residential E911 locations were also determined as the locations where emergency responders would leave the public road to gain access to a residence. In Iowa, E911 locations were created by the IGWS by first selecting all GIS parcel polygons with a site address (actual address of the property, not the owner’s address), and creating a polygon center point using GIS software for that subset of parcel polygons. Using the 2010 NAIP orthophotos as a visual reference, the location of the polygon center point was moved to the intersection of the driveway and property line. Using the parcel polygon identifier as a link, the standardized address attributes from the rooftop structure points were transferred to the E911 points. There was no offset distance from the street center line used for E911 points.

### Address-matched geocodes

AHS participant addresses were geocoded using ESRI’s ArcGIS 10 geocoding software, which uses a commercially available database of North American roads, NAVTEQ® 2011, version 2. The NAVTEQ database is regularly updated with data from local agencies which may use GPS technology as a verification tool [9]. Geocoding software was set to a street offset of 30 feet from the street center line, a squeeze factor of 10% (first and last 10% of a street segment are not used), and an address matching tolerance of 80 (reflects level of agreement needed to match an address). Addresses that were not matched to a street address (e.g., zip code, town match only) were interactively reviewed for spelling errors and format differences using the NAVTEQ reference to improve matching to the street database, or by using online map resources to manually place the point. The final group of address-matched geocodes refers to those either matched to NAVTEQ or manually placed. If discrepancies existed between these resources and additional participant information was not available, the address remained unmatched. Street addresses associated with the LH and BEEA studies were similarly geocoded, but were limited to automated geocoding procedures.

We restricted all analyses to participants whose addresses could be matched to a street address (“good” geocodes), either by automated or interactive geocoding procedures. Our main analysis dataset included Iowa participants with all three types of coordinate information: rooftop, E911, and address-matched geocodes.

### Agricultural exposures near residences

To assess the potential impacts of location error on such exposure assessments, we compared exposure classification derived from each geo-location method for two types of proximity-based environmental exposures. In a GIS, we determined whether crop fields were present or absent within a 250 and 500 meter (m) buffer of the rooftop location of the homes for four of the primary crops grown in Iowa (corn, soybean, alfalfa/hay, and other row crops combined). These distances were previously identified as probable ranges for pesticide drift [10–12]. Crop distance estimates were generated using 1990 land cover classification data derived from 1989–1991 satellite imagery. These data and distances were also used for a prior study of environmental exposure to agricultural pesticides [13].

Exposures to animal feeding operations (AFOs) were also estimated for each residential location. AFO inventories, which include information on permitted operations and estimates of animal volume and type, are publically available from the Iowa Department of Natural Resources. We determined whether one or more operative AFO (2003–2011) were present within a 2 and 5 km distance of each address. These distances were selected based on likely transport of ambient air pollution from an AFO [14].

### Measuring positional error

Positional error was defined as the Euclidean distance (m) between either the address-matched or E911 coordinates for an address and the corresponding rooftop location. For the LH and BEEA subcohorts, we compared the distance between the GPS location and the address-matched geocode; E911 and rooftop locations were not available for all addresses. We first converted all coordinates to a common projected coordinate system and datum (U.S.A. Contiguous Albers Equal Area Conic USGS version, North American Datum 1983) and then calculated these linear distances using ArcGIS (v. 10).

Descriptive statistics were generated for positional errors, overall and stratified by rural status. We estimated the prevalence of each crop type (proportion of homes with >0 crop acres) within 250 m and 500 m circular buffers around residences for each location method. We computed the sensitivity and specificity of the crop exposure metric (>0, 0 acres within buffer) using rooftop coordinates as the gold standard. Sensitivity was defined as the percentage of exposed participants (>0 acres of a crop within the buffer) that were correctly classified as exposed by the address location method. Specificity was defined as the percentage of unexposed participants (0 crop acres within the buffer) that were correctly classified as unexposed. The same approach was used to assess the validity of binary AFO exposure metrics for 2 and 5 km circular buffers around the address. To demonstrate expected attenuation of risk estimates due to exposure misclassification resulting from positional error, we estimated odds ratios (OR_obs_) for a hypothetical nested case–control study of crop exposure and cancer risk assuming a true odds ratio (OR_true_) of 2.0, using an approach described by Blair et al. [15]. In this hypothetical study, crop exposure would serve as a surrogate for agricultural pesticide exposure. We compared positional errors between Iowa and North Carolina by comparing NAVTEQ geocoded addresses to the GPS locations between the subcohorts. All statistical analyses were conducted in SAS (v. 9.3).

## Results

Among 24,527 AHS cohort participant enrollment addresses in the 14 Iowa counties (which included multiple addresses per participant and many rural route addresses that were later updated to street addresses), 14,127 (57.6%) of the addresses had good geocodes (street address geocode). Of these, 9,617 addresses were matched to a rooftop coordinate and E911 location. After removing duplicate addresses due to both an applicator and spouse participating in the study, our analysis subset included 5,064 unique Iowa addresses with both rooftop and E911 coordinates. Sixty-five percent of these addresses were located outside of town boundaries (i.e., were rural). Comparable proportions of LH and BEEA addresses were geocoded to the street address level.

The distribution of positional errors indicated that E911 locations were more accurate than the address-matched geocodes (Table 1). Median errors were smaller and the range of positional error distances were substantially narrower for E911 locations (median = 39 m; IQR = 22-61) than the address-matched locations (median = 90; IQR = 47-215). This pattern was consistent for both rural and non-rural addresses, with greater errors for rural addresses. Overall, 87% of E911 locations were within 100 m of the rooftop location; this proportion was as high as 99% for non-rural addresses and as low as 80% for rural addresses (corresponding proportions for address-matched were 53% overall, and 89% and 34% for non-rural and rural addresses, respectively).

**Table 1:** **Distribution of positional error (meters) by geo-location data sources for Agricultural Health Study addresses in 14 counties in Iowa, overall and by rural status** ^**a**^

Positional error (m)								
Geo-location data comparison	N	Min	Mean	SD	Median (IQR)	Max	Percent ≤100 m	Percent ≥1000 m
**Overall**								
Address-matched vs. Rooftop	5064	4	312	956	90 (47–215)	15171	53	6
E911 vs. Rooftop	5064	7	62	83	39 (22–61)	1276	87	0.1
**Rural** ^**a**^								
Address-matched vs. Rooftop	3296	4	444	1159	147 (78–353)	15171	34	9
E911 vs. Rooftop	3296	11	83	93	51 (39–83)	1199	80	0.1
**Non-rural**								
Address-matched vs. Rooftop	1768	6	66	151	45 (27–68)	4694	89	0.3
E911 vs. Rooftop	1768	7	23	35	19 (17–23)	1275	99	0.1

^a^Rural status was defined as a rooftop location outside of the U.S. Census 2000 Incorporated Places boundary.

The prevalence of crop fields near homes ranged from 56% (alfalfa/hay, 250 m) to 100% (corn, 500 m), and was almost always greater for rural addresses (Table 2). The sensitivity of binary crop exposures was generally high (≥80%). Sensitivities were similar for E911 and address-matched locations for the highly prevalent corn and soybean crops, and this pattern was consistent across rural and non-rural addresses. For less prevalent crops (alfalfa/hay, other), sensitivity was lower for the address-matched locations. Overall, the specificity was also high across all crop types, although it was generally better for E911 locations (e.g., 5.8 to 8.2% higher than address-matched for 250 m). Specificities for exposures to corn and soybeans were lower among rural addresses than non-rural addresses. For a hypothetical OR_true_ of 2.0, the OR_obs_ was consistently less attenuated when crop exposure was classified based on the E911 location compared to the address-matched geocode.

**Table 2:** **Accuracy of crop exposure classification for Agricultural Health Study addresses in 14 counties in Iowa by geo-location data source, overall and by rural status**

		Sensitivity		Specificity		OR_obs_^b^	
>0 Acres of crop within specified distance from home	Prevalence (%)^a^	Address-matched	E911	Address-matched	E911	Address-matched	E911
**Overall**							
Corn							
250 m	97.6	99.5	99.7	82.8	91.0	1.67	1.79
500 m	99.8	99.9	100.0	88.9	88.9	1.47	2.00
Soybean							
250 m	85.7	97.4	98.7	89.5	95.3	1.73	1.85
500 m	97.1	99.6	99.8	92.5	97.9	1.77	1.88
Alfalfa/hay							
250 m	55.7	84.9	95.4	88.8	95.8	1.62	1.86
500 m	78.5	94.8	98.9	81.9	95.2	1.64	1.91
Other row crops							
250 m	68.9	89.5	97.1	87.2	94.6	1.60	1.86
500 m	87.6	97.8	99.3	88.0	93.9	1.72	1.90
**Rural** ^**a**^							
Corn							
250 m	99.8	99.9	99.9	28.6	85.7	1.22	1.46
500 m	100.0	100.0	100.0	0^±^	0^±^	-	-
Soybean							
250 m	98.3	99.2	99.6	52.7	83.6	1.36	1.64
500 m	99.9	100.0	100.0	0^±^	50.0	-	-
Alfalfa/hay							
250 m	56.2	80.5	94.4	86.9	95.5	1.54	1.84
500 m	74.1	92.5	98.6	79.3	94.7	1.59	1.90
Other row crops							
250 m	78.2	88.4	97.0	79.6	92.2	1.44	1.79
500 m	89.8	97.4	99.5	84.0	92.3	1.63	1.91
**Non-rural** ^**a**^							
Corn							
250 m	93.5	98.7	99.3	86.1	91.3	1.69	1.81
500 m	99.6	99.8	100.0	100.0	100.0	1.50	2.00
Soybean							
250 m	62.2	92.2	96.2	92.5	96.3	1.74	1.86
500 m	91.9	99.0	99.4	93.8	98.6	1.80	1.88
Alfalfa/hay							
250 m	54.7	93.2	97.2	92.4	96.4	1.78	1.91
500 m	86.7	98.6	99.3	91.1	97.0	1.80	1.91
Other row crops							
250 m	51.6	92.4	97.3	93.6	96.6	1.80	1.91
500 m	83.7	98.6	99.0	92.7	95.8	1.85	1.89

^±^Specificity = 0 because there were no unexposed addresses.

^a^Prevalence of crops near addresses and rural status were based on rooftop locations.

^b^Where OR_true_ =2.0.

The distribution of the total number of AFOs within 2 and 5 km radii of Iowa residences did not vary between address-matched, E911, or rooftop locations, and ranged from 0 to 9 AFOs within 2 km (median = 1) and 0 to 27 within 5 km (median = 4) (data not shown). The prevalence of ≥1 AFO within a 2 km buffer of rural addresses (52.3%) was more than double the prevalence for non-rural addresses (21.7%), but was comparable within the 5 km buffer (92.0 and 91.4% for rural and non-rural addresses, respectively; Table 3). Overall, the accuracy of AFO exposure was notably higher for E911 compared to address-matched locations. The sensitivity and specificity of AFO exposure varied substantially by rural status among address-matched geocodes. Exposure sensitivity within 2 km was lower for rural (63.6%) compared to non-rural addresses (75.2%), but more similar within 5 km (94.4% and 97.2%, respectively). Specificity within 2 and 5 km was substantially lower for rural addresses (e.g., rural (67.7%) versus non-rural (84.3%) at 2 km). The improved accuracy of E911 location was most evident for rural addresses, where exposure specificity was up to 60% greater compared to address-matched geocodes. The sensitivity and specificity for E911-located AFO classifications were each >98% for both rural and non-rural addresses, resulting in little attenuation of hypothetical ORs. In contrast, the greater errors in address-matched geocodes led to substantial misclassification of AFO exposures and subsequent attenuation of hypothetical risk estimates.

**Table 3:** **Accuracy of animal feeding operations (AFO) exposure classification for Agricultural Health Study addresses in 14 counties in Iowa, by geo-location data source, overall and by rural status**

		Sensitivity		Specificity		OR_obs_^b^	
≥1 AFO within distance of address	Prevalence (%)^a^	Address-matched	E911	Address-matched	E911	Address-matched	E911
**Overall**							
2 km	41.6	65.7	98.5	75.5	99.0	1.33	1.97
5 km	91.8	95.4	99.9	48.1	99.3	1.30	1.98
**Rural** ^**a**^							
2 km	52.3	63.6	98.4	67.7	98.3	1.23	1.95
5 km	92.0	94.4	99.9	39.0	98.9	1.20	1.98
**Non-rural**							
2 km	21.7	75.2	99.2	84.3	99.8	1.46	1.99
5 km	91.4	97.2	99.9	63.8	100.0	1.49	1.98

^a^≥1 AFO present within specified distance from home was based on rooftop location.

^b^Where OR_true_ =2.0.

Within the two AHS subcohorts, there was generally greater overall error in Iowa addresses, where the median distance between the GPS reading and geocode was 131 m (IQR 65–287) compared with 99 m (53–210) for North Carolina (Table 4). A smaller proportion of Iowa geocoded addresses were within 100 m of the GPS coordinates, and there were more errors ≥1000 m than in North Carolina. This pattern held for rural addresses, where the median error was 31% higher in Iowa. Among non-rural addresses, the pattern was reversed; Iowa addresses had less error and proportionally more locations were placed within 100 m of the GPS reference location. However, non-rural addresses comprised only a small proportion of the addresses in both subcohorts (17% in Iowa and 10% in North Carolina).

**Table 4:** **Distribution of positional error (meters)** ^**a**^ **of geocoded addresses in Iowa and North Carolina from two subcohorts within the Agricultural Health Study, overall and by rural status**

				Positional Error (m)						
GPS vs. Address-Matched Geocode	N	%	Min	Mean	SD	Median (IQR)	Max	IQR	Percent ≤100 m	Percent ≥1000 m
**Overall**										
Iowa	1917	73.1	1	348	1178	131 (65–287)	28556	222	39	4
North Carolina	707	26.9	5	272	1189	99 (53–210)	20695	156	50	4
**Rural** ^**b**^										
Iowa	1583	82.6	6	406	1288	153 (84–344)	28556	260	30	5
North Carolina	637	90.1	5	262	981	105 (57–218)	20695	161	48	4
**Non-rural**										
Iowa	334	17.4	1	77	125	49 (29–82)	1673	53	81	0
North Carolina	70	9.9	9	362	2362	55 (24–112)	19830	88	71	1

^a^Positional error was determined by comparing global positioning system (GPS) coordinates taken at the entrance to the home to geocoded address coordinates using the NAVTEQ street database.

^b^Rural status was based on GPS location.

## Discussion

Our evaluation of geocoding accuracy for Iowa addresses in the AHS identified greater positional errors for rural addresses compared to non-rural addresses. In reference to a rooftop standard, E911 locations consistently had less positional error than address-matched geocodes, a pattern that held across rural status. In examining positional error of geocoded addresses between states within two AHS subcohorts, we found greater overall error in Iowa addresses compared to those in North Carolina due to the larger errors for rural Iowa addresses. The sensitivity of a proximity-based metric of exposure to corn and soybeans was not significantly impacted by the positional errors we identified, but was lower for the less prevalent crops. Specificity tended to be lower in general, especially among rural addresses. The greater positional error for address-matched geocodes compared to E911 locations led to more exposure misclassification and attenuation of hypothetical ORs for both crop and AFO exposures.

Positional error for non-rural addresses was markedly lower than for rural addresses; 89% of non-rural, address-matched geocodes were within 100 m of the rooftop coordinates, compared with only 34% of rural addresses. These larger positional errors for rural compared to non-rural geocoded addresses have been observed in other U.S. study populations [2, 5, 7, 16]. The fact that our rural E911 address locations were only slightly less accurate than the non-rural E911 locations is reassuring. We expected that E911 locations would benefit rural areas in particular, as points placed at the intersection of the public road and the residence are an improvement upon the zip code-level geocodes previously generated for many rural AHS addresses. Although based on only a sample of AHS addresses in Iowa, our data indicate that E911 locations are a substantial improvement upon address-matched geocodes for rural AHS addresses. Others have similarly identified greater accuracy in E911 locations relative to geocodes from commercial databases [6, 17]. The consequence of such positional errors will be related to the intended use of the geocodes.

The sensitivities of classifying major Iowa crops (e.g., corn and soybeans) within 250 and 500 m of homes were generally unaffected by the positional errors observed in our data. However, there were consistently (though modestly) better specificities for E911 locations compared to address-matched geocodes. We anticipated the high sensitivities found in our evaluation because over 90% of Iowa’s land use is agricultural [18]; this ubiquity of exposure is demonstrated by the similar prevalence of certain crops in non-rural and rural areas.

The impacts of geocoding error on the attenuation of ORs were most apparent in rural areas, where the median positional error for address-matched geocodes was three times as high as that for E911 locations, and when the prevalence of crops near homes was high. Use of exposure metrics based on the E911 locations generally resulted in only modest attenuation of ORs. These results are likely generalizable to other agricultural settings where the prevalence of crop exposures is very high. Our analysis of the AFO exposure metrics showed that for exposures with lower prevalence (e.g., <50%), sensitivity is disproportionately impacted by geocoding error. The increased sensitivity for AFO exposure and lower specificity as prevalence increased led to a similar attenuation bias in both buffer sizes, which was pronounced for address-matched geocodes and negligible for E911 locations.

Contrasts between the crop and AFO metric analyses suggest that the impacts of geocoding error on environmental exposure assessment are in part a function of scale, i.e., the prevalence of crops or AFOs within a specific distance. In their comparison of E911 locations versus commercial geocodes, Vieira and colleagues found that assessment of perfluorooctanoate exposure in public drinking water in West Virginia was relatively unaffected by poor geocoding accuracy, given that addresses were frequently geocoded to the correct street and the entire street shared the same water supply [6]. However, the authors cautioned that geocoding error might still result in exposure misclassification for residents with private drinking water supplies, where exposures are unique to the individual’s residence, a situation that applies to the majority of the AHS cohort. An evaluation of traffic-related air pollution exposures in an urban population in Florida found that street geocoding consistently overestimated proximity to major roads at distances up to 250 m, despite a median positional error of 41 m and few errors >100 m [19]. In the context of our findings, these studies highlight the need to consider both the prevalence and spatial distribution of exposures when judging the potential for misclassification resulting from positional error.

By comparing geocoding errors for two subcohorts within the AHS, we examined how positional errors in geocodes might differ between the two states. Our results indicated that overall and especially in rural areas, geocoded North Carolina addresses had less error than Iowa geocoded addresses. Since all BEEA and LH addresses were automatically matched, the differences in errors between the states may potentially be explained by several factors. First, improvements to the commercially available NAVTEQ database are more likely to occur in North Carolina due to its greater average population density. Rural counties with very low population densities are a low priority for NAVTEQ’s improvements of existing databases, and 25 of Iowa’s 99 counties have fewer than 20 residents per square mile [20]. Second, the topography, population distribution, and agricultural land area in Iowa and North Carolina differ substantially. The average farm size in these states is correspondingly different, with the mean and median farm size in North Carolina 168 and 51 acres, respectively, compared to 345 and 136 acres in Iowa [21]. Larger farms with multiple roadway access points and buildings may have correspondingly greater geocoding error even from automated matching if the address point is not placed near an actual residence. Notably, the LH and BEEA subcohorts were comprised of a greater proportion of rural addresses than in the entire AHS cohort, therefore our overall results likely overestimate the true error that would be observed.

In our estimation of the positional errors for both the E911 and address-matched locations, we sought to assess error based on typical uses of these data. The method of geo-locating addresses differed between these data sources. E911 data have no offset because their purpose is to aid emergency responders in finding the intersection of a driveway and public road. On the other hand, the use of an offset from the street centerline is a typical practice for geocoding with street databases. These systematic differences are unlikely to affect the interpretations of our results, however optimal offset distances could be estimated from the sample data to further reduce positional error.

Our analysis underestimated the full extent of misclassification in the AHS cohort due to the exclusion of addresses for which we could not obtain a “good” street-level geocode. We also could not estimate positional error for all Iowa counties due to the lack of rooftop coordinates or GPS locations for the entire cohort. Although E911 locations had less error than the commercial geocodes, especially in rural areas, these data were only available for 61 Iowa counties as of 2013. The assignment of E911 locations requires the use of supplementary detail from tax parcel data, digital orthophotos, and in some cases, ground identification to identify the accurate residence location; this was typically less effort than that required to obtain a rooftop GPS location. At the time of enrollment of the AHS cohort, E911 assignment was ongoing in Iowa and North Carolina, but is now largely complete. Based on our findings, it may be worthwhile to obtain E911 locations for rural addresses to reduce misclassification of GIS-based exposures in the AHS. We also note that the validity of dichotomized exposures is subject to the choice of classification cutpoints. Our buffer sizes were based on theoretical ranges for pesticide drift, but a present/absent crop exposure metric such as we evaluated is fairly crude. Alternatively, distance to the closest point of exposure as the exposure of interest or classification based on categories of acreage within these buffers may be more greatly impacted by these positional errors.

Our analyses demonstrate that positional accuracy has implications for the validity of agricultural exposure assessments that are based on GIS methods, an issue that is not unique to the AHS. Consequently, exposure misclassification is, in part, a function of the quality of commercial geocoding databases, which may vary by country. This may be of particular concern for efforts that attempt to harmonize exposure data across different studies from multiple countries. One such ongoing effort is AGRICOH, an international consortium of agricultural studies from numerous countries [22]. Any future pooling of GIS-based agricultural exposure information in such consortia should be done with an understanding of the extent and types of positional errors in each study population.

Our evaluation of positional accuracy in the AHS suggests that errors in geocodes for rural addresses may substantially impact study validity. Therefore, in the U.S. it will likely be important to obtain E911 locations for rural addresses to reduce exposure misclassification. A variety of environmental exposures with diverse spatial distributions may be examined in the AHS cohort, including environmental exposure to pesticides, ambient air pollutants, and drinking water contaminants, reinforcing the importance of this assessment. Epidemiologic studies in the AHS and other agricultural or rural study populations should carefully evaluate their geocoding approach and the resulting implications for exposure assessment in the context of study objectives.

## Electronic supplementary material

Additional file 1: Figure S1: Map of Iowa counties used in the AHS geocoding accuracy assessment. (DOC 6 MB)
