# Supplementary figures and images for: Accuracy of residential geocoding in the Agricultural Health Study
Source: Int J Health Geogr. 2014 Oct 7;13:37. doi: 10.1186/1476-072X-13-37 (PMC4203975; doi:10.1186/1476-072X-13-37)

**Additional file1: Figure S1.** Map of Iowa counties used in the AHS geocoding accuracy assessment.

**
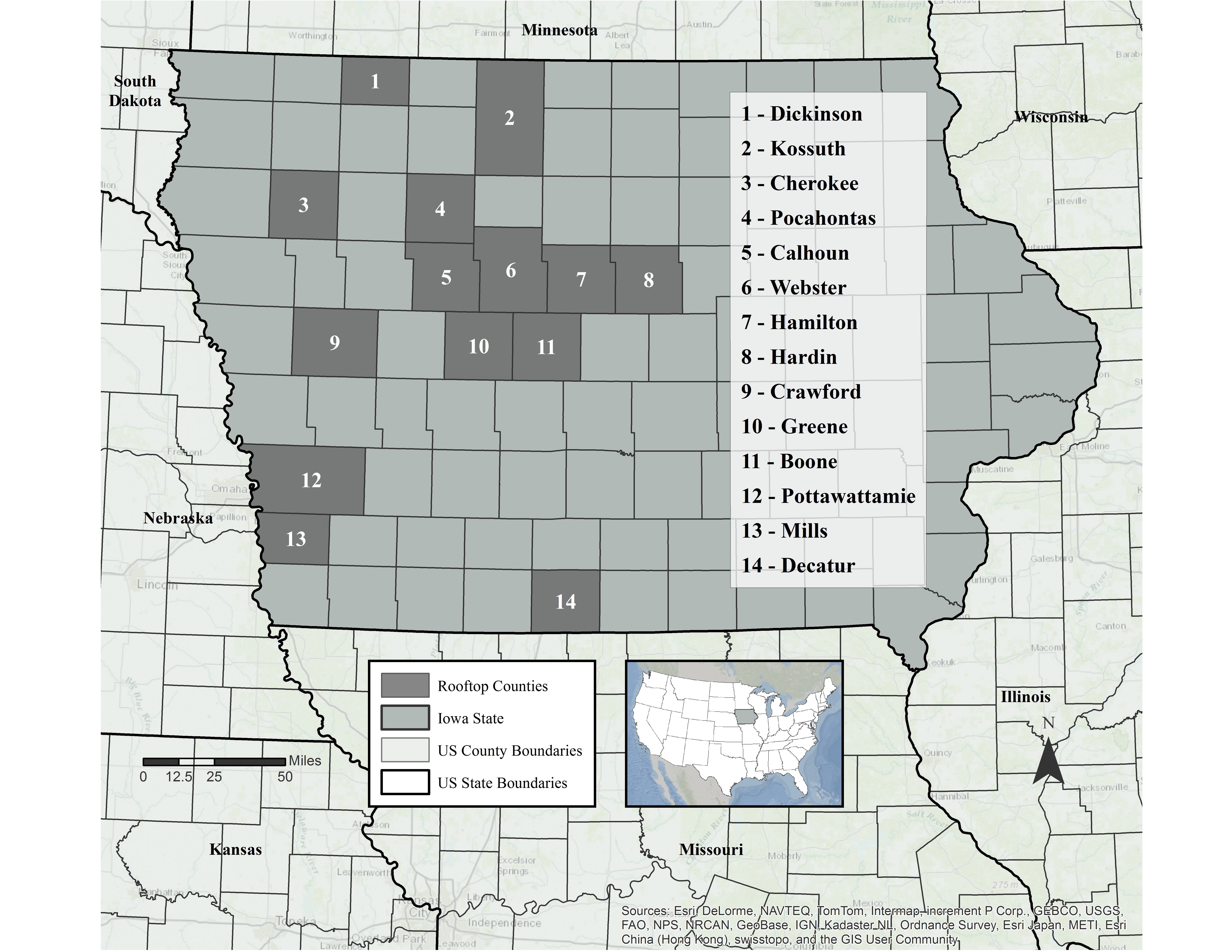
**

Supplement: Supplementary file 1 — Additional file 1: Figure S1: Map of Iowa counties used in the AHS geocoding accuracy assessment. (DOC 6 MB) [file 12942_2014_607_MOESM1_ESM.doc]
